# Ack1: Activation and Regulation by Allostery

**DOI:** 10.1371/journal.pone.0053994

**Published:** 2013-01-14

**Authors:** Ketan S. Gajiwala, Karen Maegley, RoseAnn Ferre, You-Ai He, Xiu Yu

**Affiliations:** 1 Cancer Structural Biology within Oncology Medicinal Chemistry, Pfizer Worldwide Research and Development, San Diego, California, United States of America; 2 Oncology Research Unit, Pfizer Worldwide Research and Development, San Diego, California, United States of America; University of Georgia, United States of America

## Abstract

The non-receptor tyrosine kinase Ack1 belongs to a unique multi-domain protein kinase family, Ack. Ack is the only family of SH3 domain containing kinases to have an SH3 domain following the kinase domain; others have their SH3 domains preceding the kinase domain. Previous reports have suggested that Ack1 does not require phosphorylation for activation and the enzyme activity of the isolated kinase domain is low relative to other kinases. It has been shown to dimerize in the cellular environment, which augments its enzyme activity. The molecular mechanism of activation, however, remains unknown. Here we present structural and biochemical data on Ack1 kinase domain, and kinase domain+SH3 domain that suggest that Ack1 in its monomeric state is autoinhibited, like EGFR and CDK. The activation of the kinase domain may require N-lobe mediated symmetric dimerization, which may be facilitated by the N-terminal SAM domain. Results presented here show that SH3 domain, unlike in Src family tyrosine kinases, does not directly control the activation state of the enzyme. Instead we speculate that the SH3 domain may play a regulatory role by facilitating binding of the MIG6 homologous region to the kinase domain. We postulate that features of Ack1 activation and regulation parallel those of receptor tyrosine kinase EGFR with some interesting differences.

## Introduction

Activated Cdc42-associated kinase, Ack1, belongs to one of the 10 families of mammalian nonreceptor tyrosine kinases (NRTK) [1]. NRTKs are multi-domain proteins with the catalytic activity residing in the kinase domain. A number of these families have SH2 and SH3 domains preceding the kinase domain in the protein sequence, and they may play a crucial role in the regulation of the enzyme activity. In Src- and Abl-family kinases, for example, the SH3 domain plays a pivotal role in the autoinhibition of the enzyme activity [2], [3], [4], [5]. In both these families, the SH3 domain interacts with the poly-Pro region located between the kinase and the SH2 domains, maintaining the enzyme in its autoinhibited state. Addition of SH3 domain substrates stimulates the activity of Hck, a Src family member, competing out the poly-Pro region of the enzyme and thereby releasing the autoinhibitory constraints [6]. The Ack family is unique in that it is the only one with the SH3 domain following the kinase domain in the primary sequence [7].

**Figure 1:**
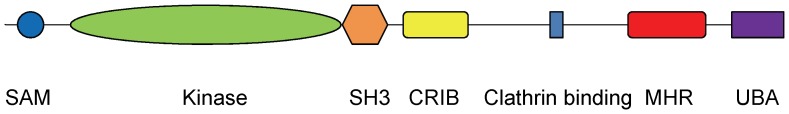
Ack1 domain architecture adapted from Prieto-Echague and Miller, 2011. SAM: Sterile Alpha Motif; CRIB: Cdc42/Rac-Interactive Binding region; MHR: MIG6 Homologous Region; UBA: Ubiquitin Association region.

The common core of the Ack family consists of the SAM domain at the N-terminus followed by the kinase domain and the SH3 domain (Figure 1). Thus the regulatory features of Ack family members are likely to be different from those of other NRTK families, for example Src and Abl, that have an SH3 domain preceding the kinase domain. In addition to the common core, Ack1 has a Cdc42/Rac-interactive binding region (CRIB), a clathrin binding motif, a MIG6 homologous region (MHR) and a ubiquitin binding domain. The largest member of its family, Ack1 was cloned by virtue of its binding to the GTP bound Cdc42 [8]. Downstream of the CRIB domain, there is a Pro-rich sequence which interacts with the adaptor protein Grb2, and via Grb2 with various receptor tyrosine kinases.

Ack1 is expressed ubiquitously, though the highest expression levels seem to be in spleen, thymus and brain, and is phosphorylated in response to a number of stimuli including EGF, PDGF, insulin and cell adhesion [9]. It has been proposed that Ack1 is responsive to multiple stimuli since Src, believed to be responsible for the phosphorylation of Ack1 activation loop Tyr284, is recruited by multiple receptor systems [10]. Though the physiological role of Ack1 is not exactly clear, it has been shown to phosphorylate p130^cas^ [11], sorting nexin [12] and Wiskott-Aldrich syndrome protein (WASP) [13]. Its amplification has been implicated in metastasis [14]. Ack1 is thought to play a role in prostate tumorigenesis [15] by activation of the androgen receptor via direct phosphorylation of tyrosine 267 [16]. More recently four somatic mis-sense mutations of Ack1 have been identified in various human cancers [17].

Biochemically measured activity of Ack1 kinase is much lower than that of other NRTKs, and autophosphorylation increases the activity only marginally [18]. Like CSK, EGFR, PASK, CHK1, GSK3b and PIM1, ACK1 does not seem to require phosphorylation to be activated. This is consistent with the structural data which suggest that the protein conformation in general, and the catalytic machinery in particular, is essentially identical in the unphosphorylated and phosphorylated states [19]. The basis of the low enzymatic activity or the regulatory mechanism of Ack1 is not yet known. There have been seemingly contradictory data as to the role of the SH3 domain in the regulation of the enzyme activity. A point mutation in the murine Ack1 SH3 domain has been reported to enhance auto-phophorylation of the tyrosine [9] but addition of a polyproline peptide, a substrate for SH3 domain, failed to activate Ack1 [18].

We report a 3.23 Å crystal structure of the Ack1 protein encompassing the kinase domain and SH3 domain. The structure provides the first glimpse of the inactive state of Ack1 which resembles the Src/Cdk-like inactive state, also adopted by autoinhibited EGFR. It shows that the disposition of the SH3 domain with respect to the kinase domain precludes it from playing a direct role in the cis-regulation of Ack1 activity. But the modeling suggests an indirect role of SH3 domain in MHR mediated autoinhibition of the kinase activity by recognition of a unique stretch of proline-rich sequence of MHR. The structure reveals the unusual constitution of the ligand binding site of the SH3 domain. We also present a high resolution structure of the isolated kinase domain. Like the other known crystal structures of the Ack1 kinase domain, the protein is in the active conformation and shows the same dimeric interactions in the crystal lattice. The arrangement of the two molecules in the asymmetric unit provides a hitherto unnoticed hint of the role of dimerization in the kinase activation. It has been proposed that the N-terminal SAM domain mediates self association and subsequently increases kinase activity in cells [20]. Consistent with this, we have observed that dimeric GST-tagged Ack1 is significantly more active than non-tagged enzyme in biochemical studies. Together these data suggest a role for dimerization in the activation of the Ack1. We hypothesize that the arrangement of the protein dimer in the crystal lattice is reflective of the solution state of the active Ack1 kinase domain. With the available structural and biochemical data we propose that monomeric Ack1 kinase is in its autoinhibited state; allosteric interactions, *a la* EGFR, may play a major role in the regulation of Ack1 kinase activity.

## Results and Discussion

### Activated Kinase Domain and Crystal Packing

**Figure 2:**
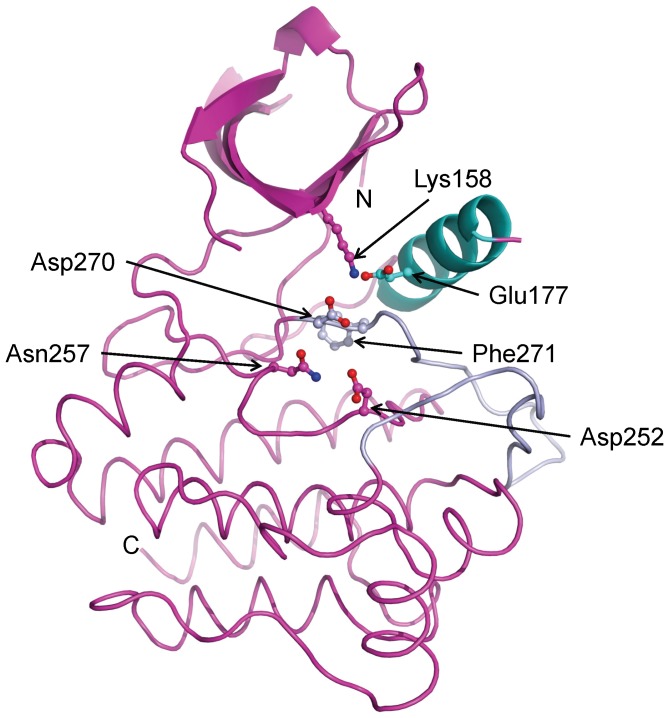
The activated state of the Ack1 kinase domain. For clarity, secondary structure for only the N-terminal lobe is displayed. The activation loop is shown in light blue and the αC helix in cyan. Functionally important residues are highlighted.

There have been five crystal structures of the Ack1 kinase domain reported previously, all of which show the protein in its active conformation irrespective of its phosphorylation state [19], [21]. We determined a 1.3 Å structure of the Ack1 kinase domain spanning residues 115–389 (Table 1). The structure is very similar to the previously reported structures with two molecules per asymmetric unit. The Ack1 kinase domain is a bi-lobed structure shared by most other eukaryotic protein kinases with the N-terminal lobe composed of a β-sheet and a catalytically important αC helix, and a larger C-terminal lobe that is predominantly α-helical (Figure 2). The inter-lobe cleft has evolved for ATP recognition. In its active state, the conserved Asp-Phe-Gly motif is oriented such that Asp is facing the ATP binding pocket and, along with Asn257 can coordinate two divalent cations, while the Phe sidechain is buried in the hydrophobic core (Figure 2). Asn257 also engages and orients Asp252 to begin nucleophilic attack on the hydroxyl of the incoming substrate. The A-loop takes on a well defined mostly extended conformation that creates the substrate binding site. Conserved Lys158 and Glu177 residues from the αC helix form an ion-pair interaction (Figure 2). The polypeptide chain from residue 135 to 138 belonging to phosphate/nucleotide-binding P-loop is disordered, and so is the loop connecting the N-terminal β-sheet to the catalytically important αC-helix, as is often the case for the activated conformations of other protein kinases.

**Table 1:** Crystallization, data collection and refinement.

	Ack1 kinase domain	Ack1 kinase+SH3
Crystallization conditions	22–24% PEG 3350, 0.2 M Ammonium sulfate, 0.1 M bis-tris pH 6.6, 10–20mM TCEP (pH 7), 13°C	12–15% PEG 400, 0.1 M Bicine pH 9, 25 mM TCEP (pH 7)
Data collection		
Beamline	APS 17ID	APS 17ID
Resolution	1.31	3.23
Space group	P2_1_	P4
Unit cell parameters (Å, ^o^)	71.1, 42.3, 92.6; β = 98.6	145.5, 145.5, 103.7
Contents of the asymmetric unit	Two kinase domains	Four kinase+SH3 domains
Measured reflections	472,369	227,669
Unique reflections	124,856	34,878
Data redundancy	3.7	6.5
Data completeness (%)	97.7 (93.6)	99.5 (96.7)
R_merge_	0.107 (0.581)	0.187 (0.477)
I/σ	11.2 (2.3)	9.9 (2.0)
Refinement		
R_work_/R_free_ (%)	19.5/20.9	24.6/27
Number of protein atoms	4,270	10,200
Number of non-protein molecules	496 water, 2 SO_4_^−2^, 2 ethylene glycol	0
RMSD, bond lengths (Å)	0.003	0.009
RMSD, bond angles (^o^)	0.8	1.1
PDBID	4HZR	4HZS

**Figure 3:**
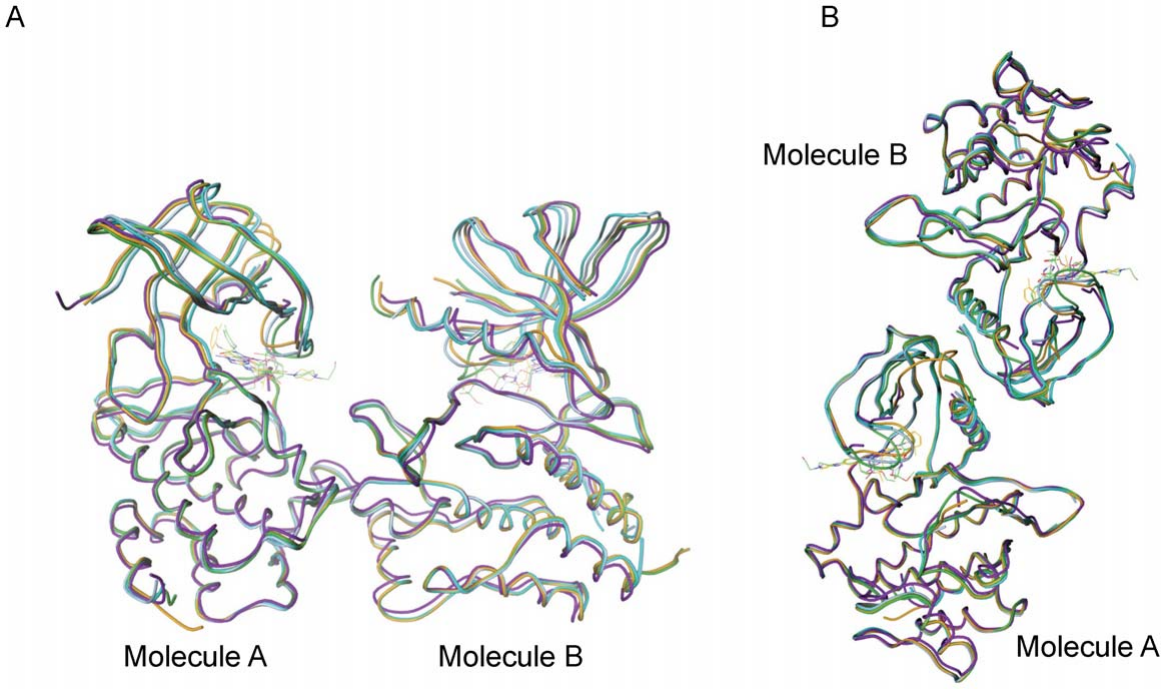
Asymmetric units of known structures of Ack1 kinase domain. (PDBIDs: 1U4D in blue, 1U46 in cyan, 1U54 in magenta, 3EQP in green and 3EQR in orange) [19], [21]. (A) Non-crystallographic dimers as deposited in the PDB. (B) An alternative non-crystallographic dimer proposed to be of biological relevance.

**Figure 4:**
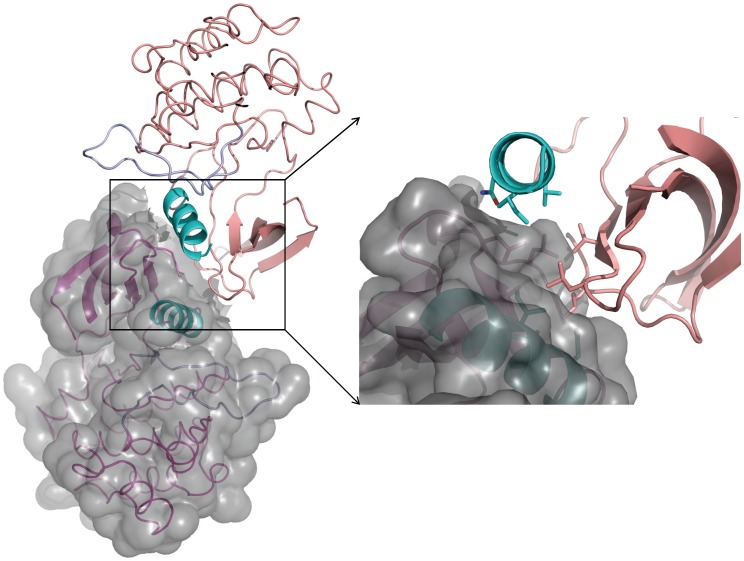
Symmetric dimer interface of active Ack1 kinase domain. The solvent accessible surface of molecule A is depicted. The right panel shows the zoomed view of the interface between the two molecules. The same sidechains (Leu120 from β1, Ile175, Val178 and Asn179 from αC, Val195 and Leu197 from β4) from the two molecules participate in reciprocal interactions. These are mostly non-specific interactions, except for Asn179 sidechain, which is within hydrogen bonding distance from the backbone carbonyl oxygen (not shown) of Leu197 from the dimeric partner.

All the known crystal structures of the Ack1 kinase domain belong to four different crystal forms and yet all, including the one reported here, display the same packing of the two molecules in the asymmetric unit. These molecules could be arranged in two potential pseudo-symmetric non-crystallographic dimers. The asymmetric units of the structures deposited in the PDB show a dimer where the two kinase domains face each other (Figure 3A). An alternative arrangement is the one where the pseudo 2-fold axis packs the N-terminal lobes of the two molecules in a head-to-head symmetric dimer (Figure 3B). The compelling aspect of this alternative arrangement is that it suggests a model where activation of Ack1 is achieved through dimerization and is independent of its phosphorylation state, much like EGFR and CDK/cyclin models. The formation of the Ack1 symmetric dimer is mediated by the hydrophobic patch near the αC helix, burying about 1100 Å^2^ at the interface. The hydrophobic patch close to the αC helix of one molecule is packed symmetrically against the same region of the other. The N-terminal region, the catalytically important αC-helix, and the loop between strands β4 and β5 from one monomer are symmetrically packed against the same elements from its dimeric partner (Figure 4). The amino-acids at the interface are nearly exclusively hydrophobic, and this hydrophobic patch is conserved among Ack family members. In some aspects, this is reminiscent of the epidermal growth factor receptor (EGFR) situation.

EGFR, a receptor tyrosine kinase, does not require phosphorylation of the kinase domain in order to be activated. Instead, activation of EGFR is via asymmetric homodimerization of the kinase domain. Early clues to this finding came from the observation that a unique arrangement of EGFR kinase domains in active state repeated itself in majority of structures, irrespective of the crystal form. Eventually, cleverly designed experiments showed that the intracellular kinase domain of EGFR functions as an asymmetric dimer [22]. By virtue of the packing of the N-terminal lobe of one molecule against the C-terminal lobe of its dimeric partner, it is an asymmetric dimer. Activated Ack1 kinase domain on the other hand is observed in the crystal lattice as a symmetric dimer. Despite different geometric arrangements of the EGFR and Ack1 dimers, the region of the N-lobe involved in dimerization is nearly identical in both kinases.

To evaluate the role of dimerization in the activation of Ack1, we determined the k_cat_ and K_m_^ATP^ values of four protein constructs: kinase-SH3-CRIB spanning residues 110–476 with and without GST tag, kinase-SH3 spanning residues 115–453 and the isolated kinase domain spanning residues 115–389. All four protein constructs were assayed in their phosphorylated and unphosphorylated states (Table 2). What is most interesting from the biochemical data is that the GST tagged protein shows a 20–30 fold higher k_cat_ value than that for its untagged counterpart or the isolated kinase domain. GST exists as a dimer in solution [23], has been observed to cause the self association of proteins when expressed as a fusion construct [24] and would be expected to promote dimerization of the GST-kinase-SH3-CRIB construct. Based on the model proposed above for the activation of the Ack1 kinase, GST-tagged construct would be expected to show the highest level of activity, as the biochemical data show.

**Table 2:** Kinetic characterization of Ack1.

Enzyme^a^	k_cat_ (min^−1^)	K_m_ (ATP, µM)	k_cat_/K_m_(µM^−1^ min^−1^)
GST-CD	515±140	240±90	2.1
P-GST-CD	332±13	120±70	2.8
CD	26±2	140±30	0.19
P-CD	65±2	210±40	0.31
KD	16.5±0.6	134±9	0.12
P-KD	32±3	177±9	0.18
KD+SH3	10±0.3	140±10	0.07
P-KD+SH3	22±2	181±4	0.12

a GST-: GST-tagged protein; P-: phosphorylated protein; CD: residues 110–476 spanning kinase domain, SH3 domain and CRIB region; KD: residues 115–389 spanning the kinase domain; KD+SH3: residues 115–453 spanning kinase and SH3 domains.

Values are averages ± SD.

The largest gain in catalytic efficiency upon phosphorylation, as measured by k_cat_/Km value, is 1.7 fold, all of which comes from higher k_cat_ value for the phosphorylated form of theenzyme. These results are consistent with previous reports suggesting that phosphorylation does not greatly enhance the enzyme activity of Ack1 [18]. We hypothesize that the concentration dependent dimerization of the Ack1 kinase domain in the crystal lattice may be mimicking the physiological situation of SAM domain mediated association of Ack1. Such self association has been shown to increase autophosphorylation of the kinase domain in cells [20]. The dimerization constant of the isolated kinase domain is unlikely to be strong enough to form dimers in solution; isolated Ack1 kinase domain is monomeric at the sub-millimolar concentrations attained during size exclusion chromatography (data not shown). Crystallization of the protein increases the concentration over two orders of magnitude from about 120 µM in solution to 24 mM in the crystal lattice, facilitating dimer formation possibly causing the activated conformation seen in the crystal structure.

**Figure 5:**
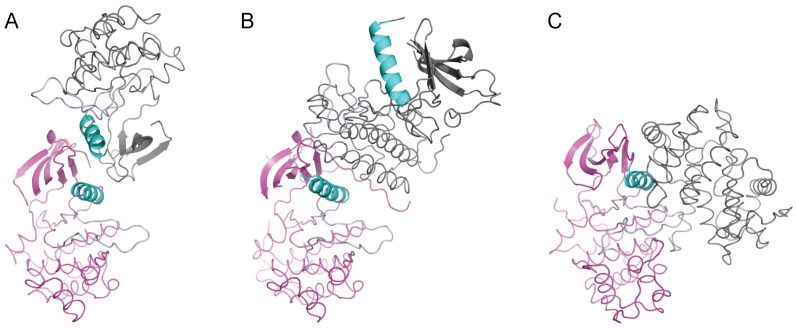
Allosteric activation of Ack1, EGFR and CDK. Allosteric activation via (A) symmetric dimerization of Ack1, (B) asymmetric dimerization of EGFR and (C) heterodimerization of CDK2-cyclinA. Backbone trace of each molecule of the dimeric complex is drawn, highlighting the αC helix of each kinase domain in cyan and the activation loop in blue. Molecule A of each kinase domain is shown in magenta and molecule B (cyclin in case of CDK2) in gray. For clarity, the secondary structure of only the N-terminal lobe is displayed.

The proposed allosteric activation of Ack1 shares some similarities with CDK2 and EGFR (Figure 5), and yet each one is unique in its own way. Whereas CDK2 activates through heterodimeric interaction with cyclin A [25], EGFR and Ack1 are activated by homodimeric interactions. In case of EGFR, it is the asymmetric homodimer that is the active form, while Ack1 forms symmetric homodimer. Whereas EGFR activation does not require phosphorylation for activation, CDK2 gains only the basal activity upon binding with cyclinA; phosphorylation of the A-loop Thr fully activates it. Ack1 kinase domain on the other hand seems to have some basal activity in the solution state, which increases marginally upon phosphorylation. The presence of the GST-tag (or the SAM domain [20]) has a much bigger impact on the enzyme activity, likely by promoting dimerization.

### Inactive State of the Ack1 Kinase Domain in Kinase+SH3 Construct

**Figure 6:**
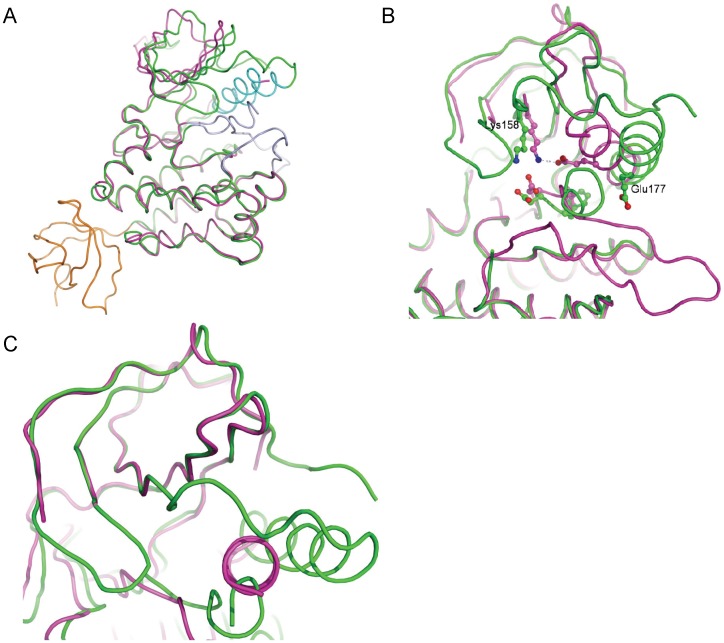
Differences and similarities between the inactive and active states of Ack1 kinase domain. The inactive kinase domain is shown in green, the SH3 domain in orange and the active kinase domain in magenta. (A) Overall superposition of the kinase domain in the two states show the different orientations of the N-lobes relative to the C-lobe. RMSD of the Cα positions of the C-lobe residues 292–389 is 0.6 Å between the two states. The αC helices are highlighted in cyan and the activation loops in light blue. (B) Close-up of the ATP-binding pocket shows the changes accompanying the activation of the kinase domain. The beginning of the helical A-loop can be seen in the inactive state, while in the activated state, it is in the extended conformation. The Lys158– Glu177 ion-pair of the activated state is broken, and the αC helix is rotated away from the ATP-binding site in the inactive state. (C) Superposition of the N-terminal lobe shows that the overall conformation, except the αC helix, changes little between the two states. The β-sheet and α-helix move independently as rigid bodies relative to the C-lobe through the catalytic cycle.

The 3.23 Å crystal structure reported here spans Ack1 kinase and SH3 domains (residues 115–453) (Figure 6). There are four molecules per asymmetric unit (Figure S1). The structures of the four monomers in the asymmetric unit are essentially identical. It gives the first glimpse of the Ack1 kinase domain in its inactive state which resembles the Src/CDK like inactive state also seen in other receptor tyrosine kinases including EGFR.

Residues 115–446 are modeled, with the exception of residues 279–291, for each of the four molecules. There is no appreciable electron density to model the activation loop region 279–291 for any of the molecules in the asymmetric unit. However, the N-terminal end of the activation loop shows strong electron density to model just under two turns of the helix, which is one of numerous structural features that seem to stabilize this inactive conformation of the enzyme. Helical beginning of the activation loop following the DFG-motif prevents the αC helix from moving into its catalytically competent state. Leu273 (immediately following the DFG-motif), Ala276 and Leu277 contribute to the hydrophobic cluster in this region, parts of which are contributed by the G-loop (Phe137), the rest of the β-sheet (Leu160, Met203, Leu273, Leu192, Leu195, Met201, Leu197) and the αC-helix (Phe174, Val178, Met181). In the process, they stabilize the outwardly rotated conformation of the helix, maintaining it in the catalytically incompetent state. The interaction between the ion-pair Lys158 and Glu177, highly conserved among active kinases, is broken. Glu177 instead makes an alternate ion-pair interaction with Arg275 of the α-helical part of the A-loop, contributing to the stability of the outwardly rotated conformation of the αC-helix.

**Figure 7:**
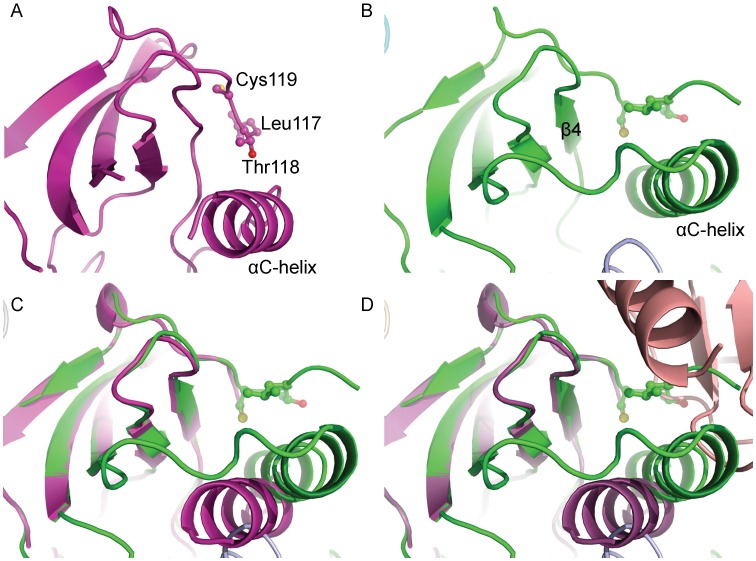
Altered packing of the N-terminal end in the inactive state precludes dimerization and consequent activation. The region around the hydrophobic patch is highlighted for (A) active state and (B) inactive state. Same residues are highlighted in both panels. (C) Superposition of the N-terminal lobe in the active (magenta) and the inactive (green) states highlights the local reshaping of the hydrophobic patch due to the movement of the αC-helix. The area of the activated molecule involved in dimer formation is shown in surface representation. (D) Superposition of N-lobe of the inactive state (green) on the N-lobe of the molecule A (magenta) of the active dimer clearly shows that the N-terminal stretch of the protein in the inactive conformation (green) occupies the same region as that by the dimeric partner (salmon) in the activated state.

Other than the differing conformations of the activation loop, the activated and the inactive states of the enzyme differ from each other by virtue of two independent rigid body rotations in opposite directions relative to the C-terminal lobe (Figure 6). One of these involves the rotation of the N-terminal β-sheet. Pivoted around the gatekeeper Thr205 residue, the β-sheet is rotated in the activated kinase domain relative to the C-terminal lobe. The second rotation is that of the αC helix about residue Leu184 in the opposite direction. Taken together, repositioning of the αC helix relative to the β-sheet obliterates the surface features that may be important for the dimerization and activation of the kinase domain (Figures 6 and 7), and leads to the misalignment of the catalytically important residues in the inactive state.

Besides the broad differences between the activated and the inactive states of the Ack1 kinase domain, there is a subtle difference between the main chain trace of the N-terminal region of the kinase domain in the two conformations (Figure 7). In the active state, the N-terminal region is pointing toward the C-lobe, away from the N-lobe of the protein. Leu117 at the N-terminus is surface exposed, Thr118 is buried between αC and β4, and Cys120 is mostly buried under β2 (Figure 7A). In the inactive conformation, Thr118 is surface exposed and Leu117 and Cys119 are buried, engaging the hydrophobic patch between αC and β4 (Figure 7B), thereby stabilizing the inactive conformation of the enzyme. This also prevents the kinase domain from forming the dimer required for activation without significant structural reorganization. This may be of physiologic significance considering that kinase domain is separated from the upstream SAM domain by ∼40 residue polypeptide chain. Interaction between SAM domains from two Ack1 molecules would increase the local concentration of the kinase domain, inducing a conformational change, promoting their dimerization and facilitating the enzyme activation. Different interaction partners for the SAM domain, could result in different orientations of the linker region thereby influencing the conformation of the N-terminal extension of the kinase domain and promoting or preventing the dimerization that would determine the activation status of the kinase domain.

### SH3 Domain and It’s Role in Regulation of Activity

**Figure 8:**
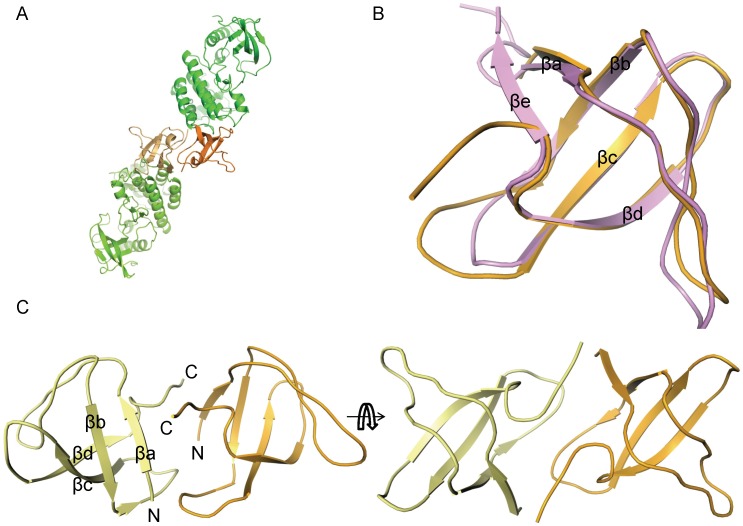
SH3 domain mediated dimer of the Ack1. (A) The kinase domains are highlighted in green and the SH3 domain in orange. Different shades are used for the individual molecules. Over 1500 Å^2^ of surface area is buried between SH3 domains from two molecules. (B) Overlay of the Lck SH3 domain (pink) on Ack1 SH3 domain (orange). (C) Symmetric packing of two SH3 domains in two orthogonal views.

In terms of the sequence, the closest relative of the Ack1 SH3 domain with a known structure is the Stam2 SH3 domain sharing 42% sequence identity. Structurally, it resembles the Lck SH3 domain (PDBID: 2iim) with 1.15 Å RMSD between 52 equivalent Cα atoms (Figure 8). A typical SH3 domain has five β-strands, βa - βe, [26] separated by four loops: RT-loop, n-Src loop, distal loop and 3_10_ loop that has 3_10_ helical character. Two antiparallel β-sheets, one made by βb, βc and βd packing orthogonally against the other formed by the N-terminal βa, βb and C-terminal βe make up the five stranded β-barrel architecture. Overall, the Ack1 SH3 domain shares this fold, except that it has a four stranded β-barrel. The last part of the polypeptide chain corresponding to βe strand turns away from βa (Figure 8). SH3-SH3 contacts between the neighboring molecules seem to preclude the interaction of the C-terminal end with the βa strand.

Each SH3 domain is involved in intermolecular contact with another SH3 domain from a neighboring molecule, mutually burying about 1500 Å^2^ (>20%) of the exposed surface area, which is the largest single region buried in this crystal form (Figure 8A). Considering the extent of the intermolecular interface, it is possible that these interactions mimic some physiologically relevant interaction (either with self or with a different SH3 containing partner protein) in the cellular environment. The lack of conservation of interfacial residues even among Ack family suggests that such an interaction, even if physiologically relevant, would be specific for the human Ack1 SH3 domain.

Another face of the SH3 domain is intimately packed against the distal face of the C-terminal lobe of the kinase domain burying in excess of 800 Å^2^ between the two. With the absence of a flexible linker between the two domains, the number of relative orientations that they can adopt is likely to be rather limited. The SH3 domain does not come in direct contact with the activation loop or the αC helix, suggesting that it does not directly influence the kinase activity of Ack1. Biochemical data further show that the presence of SH3 domain does not influence the kinase activity (Table 2), underscoring that it may not play a direct role in regulating enzyme activity. But an indirect role in the regulation of the enzyme activity cannot be ruled out. For example, it has been suggested that the SH3 domain may play a regulatory role in Ack1 kinase activity via its interaction with Pro rich regions of the protein [9].

**Figure 9:**
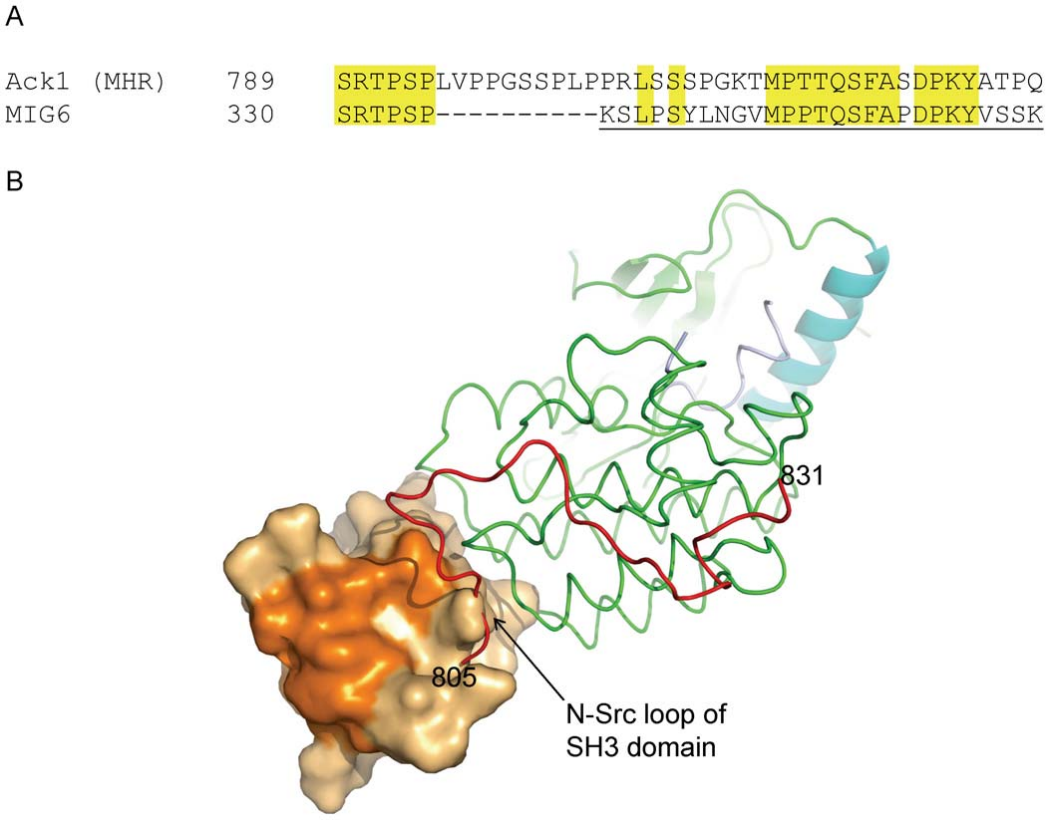
Modeling of MHR interaction with Ack1. (A) Partial sequence alignment of the MIG6 sequence with its homologous region in Ack1 protein. The residue identities are highlighted. Emphasis is on the part of the MIG6 sequence that has been modeled in MIG6-EGFR complex structure [27]. MIG6 residues present in the model are underlined. (B) The MIG6 fragment (corresponding to residues 805–831 of MHR) present in EGFR is modeled onto the inactive state structure of Ack1 kinase domain+SH3 domain structure. The SH3 domain is shown in surface representation. The substrate peptide binding site, as surmised from other SH3-peptide complex structures, is highlighted in deep orange shade. The MIG6 fragment, as bound to EGFR kinase domain, would present a steric conflict near its N-terminal end with the SH3 domain, as can be seen in the figure.

Downstream of the SH3 domain, Ack1 has a MIG6 homology region (MHR). MIG6 is a feedback regulator of ErbB family kinases. The MIG6 recognition region of EGFR has close sequence and structure resemblance to the corresponding region of the Ack1 kinase domain, and MHR has been proposed to bind and inhibit the Ack1 kinase domain in an analogous fashion [20], [27]. Based on the EGFR+MIG6 structure [27], a model for the MHR recognition can be built using the Ack1 kinase domain+SH3 domain structure reported here. In such a model, the N-terminal residues of the kinase domain recognition segment of MHR are positioned proximal to the substrate binding site of the SH3 domain and present steric conflict with its n-Src loop (Figure 9). This suggests that this region of MHR must reorient (relative to the corresponding region in MIG6+ EGFR co-crystal structure) to interact with the SH3 domain.

Just upstream of the kinase recognition segment of the MHR, there is an insertion of ten residues (relative to the MIG6 sequence) (Figure 9A). This insertion is rich in Pro residues and, based on its size, location and juxtaposition of the SH3 domain relative to the kinase domain, is likely to constitute SH3 domain interacting motif. By interacting with the SH3 domain, it may provide additional stability to MHR – kinase domain complex, inhibiting the enzyme activity during its quiescent state. The absence of the SH3-interaction motif in MIG6 is consistent with the absence of SH3 domain in its target enzymes, the ErbB family members. The substrate binding site of the SH3 domain suggests “minus” binding mode of its cognate ligand [28], which is consistent with the expected recognition of MHR, based on MIG6-EGFR structure.

The substrate binding site of the Ack1 SH3 domain is quite unique, and could be expected to undergo some changes upon substrate recognition. Based on the known structures of other SH3 domains in complex with their cognate ligands, we identify Gln397, Glu401, Lys404, Ile416, Tyr423, Trp424, Arg426, Pro437, Arg440 and Asn441 as the residues constituting the surface of the SH3 domain where the substrate is expected to bind (Figure 9B). This substrate binding surface is almost completely accessible in the crystal structure. The presence of Lys404, Tyr423 and Pro437 makes the binding site unusual. No other SH3 domain of known structure has Lys404 and Pro437 at the structurally equivalent position. An overwhelming majority of SH3 domains that have been structurally characterized show an acidic residue at the position equivalent to Lys404 in the RT loop, which may play a role in providing specific interaction with the bound substrate. For example, in the SH3 domain of p40^phox^, the structurally equivalent Glu188 is seen making ion-pair interactions with the Arg of the substrate [29]. The presence of Lys404 may suggest a different mode of substrate recognition. Tyr423 is another residue that is unusual for its location. There is only one other instance of an SH3 domain where an aromatic residue is seen at the equivalent position [30]. Comparison with the known SH3-peptide complexes suggests that Tyr423 in Ack1 SH3 domain occludes the binding site. Its sidechain must take a different rotamer if the substrate is to bind in a conventional fashion.

Trp424 is the most highly structurally conserved residue among SH3 domains of known structures. It is surface exposed and is involved in van der Waal’s contacts with the poly-Pro ligand in substrate bound SH3 domain structures. Likewise, in the Ack1 kinase+SH3 structure it is highly exposed and expected to interact with the substrate peptide. Its mutation to Lys has been observed to increase autophosphorylation in cells [9]. The structure suggests that the substitution could compromise substrate recognition, undermining possible autoinhibition by MHR and increasing autophosphorylation.

A single somatic mutation, M409I, located in the SH3 domain of Ack1 has been identified in gastric adenocarcinoma. In the structure presented here, the site of mutation is surface exposed, and though the structurally equivalent residue is not seen interacting with SH3 domain substrates in other structures, it is proximal to the substrate binding site. This suggests that Met409 could be involved in the recognition of the poly-Pro region of Ack1 which may bring MHR in contact with the kinase domain to play its negative regulatory role.

**Based on the foregoing discussion, we speculate that the 10 residue insertion in the MHR** (relative to MIG6) may interact with the SH3 domain and orient MHR for inhibitory interactions with the kinase domain, stabilizing an auto-inhibited state. This hypothesis would explain why a protein construct lacking the MHR region could not be activated by SH3 substrate peptides. The kinase-SH3-CRIB construct that was used for the study [18] lacks the internal regulatory element that could be competed out using a SH3 substrate peptide, and hence failed to show enhancement of the activity. The failure of the full-length enzyme to show any activation upon the addition of the SH3 peptide may have to do with the peptide used; it lacked sequence similarity with the MHR segment that, we suspect, interacts with the SH3 domain (Figure 9) and hence failed to compete it out. Enhanced autophosphorylation of the W424K mutant [9] on the other hand is consistent with our model. As mentioned before, Trp424 is highly conserved and forms part of the substrate binding site of the SH3 domain. Its mutation to Lys would adversely impact autoinhibition by weakening the recognition of MHR by SH3 domain.

**So the question is why does the protein construct encompassing the kinase domain and SH3** domain crystallize in the inactive state whereas the isolated kinase domain crystals always show the protein in the active state? We believe that the characteristic of the crystal packing may have allowed the protein to retain its inactive solution state even at high concentration in the lattice. As mentioned above, the extent of interfacial area buried at the SH3-SH3 interface in the crystal lattice is larger relative to the burial of the N-lobes in the activated dimeric structures of the kinase domain constructs. Based on this observation we believe that the SH3 domain mediated dimerization may be stronger than the N-lobe mediated interaction in the kinase domain+SH3 domain protein construct. Hence during the crystallization as the local protein concentration increases, SH3-domain mediated dimers may form and nucleate the crystal lattice before the N-lobe mediated activated dimer can form. Consistent with this hypothesis is the observation that the protein concentration in crystal lattice of the inactive form is about half that in the crystal lattice with the active kinase domain.

### Regulation of the Kinase Activity and Its Similarities with EGFR

We propose that the mechanism of regulation of the kinase activity in Ack1 largely parallels the model developed for EGFR [31], though EGFR is a receptor tyrosine kinase while Ack1 is a NRTK. Both enzymes have low inherent activity under generic assay conditions, and their activities are independent of the phosphorylation state. The EGFR kinase domain is preceded by the juxtamembrane domain which plays a crucial role in the dimerization and hence in the activation of the enzyme [32], [33]. The absence of a juxtamembrane domain adversely impacts the dimerization constant and the kinase activity. The Ack1 kinase domain, on the other hand, is preceded by a SAM domain. There has been experimental evidence showing that SAM-mediated dimerization increases the Ack1 kinase activity [20] and the deletion of the SAM domain adversely impacts the enzyme activity [9] suggesting the SAM domain in Ack1 may be playing a role analogous to the juxtamembrane domain in EGFR activation. Crystal structures of the isolated kinase domains of both enzymes show their activated states due to the concentration dependent activation of the enzyme. Based on the hypothesis we propose, the Ack1 kinase domain at low concentration is in autoinhibited state. The inactive state of the Ack1 kinase domain seen in the crystal structure may represent the quiescent autoinhibited state, which very closely resembles the autoinhibited state of the EGFR kinase domain [22].

One significant difference between EGFR and Ack1 kinase domains is the mode of dimerization; while activation of Ack1 is through symmetric dimer, that of EGFR is mediated by asymmetric dimerization.

## Materials and Methods

### Cloning, Expression and Purification of Protein

The Ack1 kinase domain containing residues 115–389 with N-terminal His6 tag and TEV protease cleavage site was subcloned into pFastbac vector and expressed in SF21 cells (acquired commercially from Expression Systems, LLC). The pellet was resuspended in 2X volume to weight lysis buffer (25 mM Hepes, pH 7.5, 10% Glycerol, 0.3 M NaCl, 1 mM NaVO4, 10 mM NaF, 0.5 mM TCEP, 20 mM Imidazole, 1× protease inhibitor (Roche) w/o EDTA) and microfluidized twice to lyse the cells. Triton X-100 was added to a final concentration of 0.1%. The lysate was centrifuged at 40,000 rpm for 30 min (Beckman ultra) at 4°C to obtain the soluble fractions. The supernatant was incubated with Ni-NTA agarose at 4°C for 2 hr. The Ni-NTA agarose was packed onto a column, washed, and eluted with 400 mM Imidazole. His6-TEV enzyme was added at 1:100-1:25 molar ratio. The solution was dialysed overnight at 4°C against 25 mM Hepes, pH 7.5, 10% Glycerol, 0.3M NaCl, 20 mM Imidazole, 0.5 mM TCEP. The His tag and His tagged-TEV protease were removed by passing through Ni-NTA agarose and collect the flow through. The flow through fractions were collected, concentrated and purified further on a Superdex 100 column equilibrated with 25 mM Hepes, pH 7.7, 10% Glycerol, 0.3 M NaCl, 0.5 mM TCEP, 20 mM MgCl2. The final protein was concentrated to 3–6 mg/ml and stored at −80°C.

The Ack1 kinase+SH3 domain containing residues 115–453 was cloned and purified similarly as the kinase domain. The final buffer is 25 mM Hepes, pH 7.5, 20 mM MgCl2, 0.5 mM TCEP, 0.3 M NaCl, and 10% Glycerol.

Unphosphorylated GST tagged Ack1 (residues 110–476) was purchased from Life Technologies; catalog number PV4807.

### Biochemical Assay

Ack1 kinase activity was monitored continuously using a lactate dehydrogenase/pyruvate kinase coupled assay system. 12.5–200 nM Ack1 was incubated in assay buffer (25 mM HEPES, 25 mM MgCl_2_, 1 mM TCEP, 0.001% Tween-20, pH 7.5) containing various concentrations of ATP and peptide (KVIYDFIEKKKKG) in a total volume of 160 µL (Figures S2 and S3). All assay components except enzyme were incubated at 37°C for 10 minutes. The reaction was initiated by the addition of enzyme. The reaction progress curve was followed for 30 minutes and initial velocity was estimated by fitting a portion of the curve (typically 2–20 minutes) to a straight line. For k_cat_ and K_m_ measurements the concentration of ATP was varied as appropriate. Active enzyme concentration for all enzymes studied was determined by active site titration using a tight binding inhibitor with the exception of the pGST enzyme.

### Crystallization, Data Collection, Structure Solution and Refinement

Purified Ack1 kinase domain and kinase domain+SH3 domain were crystallized using the hanging-drop vapor diffusion method at 13°C. For the kinase domain, 2 µl drops were set up on siliconized glass coverslips in a 1:1 ratio of protein to well reservoir solution containing: 0.1 M Bicine pH 9, 12–15% PEG 400, 25 mM TCEP that had been pre-titrated to pH 7.0 with Tris/HCl. Crystals grew as plates and reached data quality size (0.3×0.3×0.1 mm) in approximately 5 days. kinase+SH3 was set up similarly, with 2 µl drops in a 1:1 ratio of protein to well reservoir solution containing: 0.2 M AmSO4,.1M Bis-Tris pH 6.6, 22–24% PEG 3350, 10–20 mM TCEP pre-titrated to pH 7.0. Crystals grew as long, thin rods, and reached a size of 0.3×0.05×0.05 mm in approximately 7 days. Crystals from both constructs were soaked in a solution of their corresponding mother liquor containing 22% glycerol prior to being frozen in liquid nitrogen.

X-ray data for the Ack1 kinase and kinase+SH3 domain crystals were collected on IMCA-CAT 17ID beamline at the APS. Data were processed using autoPROC.

Ack1 kinase domain crystals were isomorphous with the published structure (PDBID: 3EQR), which was used as the starting model and refined using the program CNX.

The kinase+SH3 domain structure was solved by molecular replacement using the program phaser in CCP4. There are four molecules in the asymmetric unit of these crystals. The C-lobes of all four kinase domain were first located by molecular replacement. The N-lobes were next built-in manually in the four-fold averaged Fo-Fc difference maps. Using improvement in the phases with four kinase domains modeled, three of the SH3 domains were placed in the final stage of the molecular replacement using phaser; the SH3 domain of Hck (PDBID: 1qcf) was used as the search model. Non-crystallographic symmetry was employed to place the SH3 domain of the fourth molecule. The model was refined using CNX.

### Structure Analysis

Structural similarities of the kinase domain and the SH3 domain were analyzed using the program CE [34]. The program conducts a binary comparison of given two protein structures and provides structure based sequence alignment, sequence identities as well as structure similarity scores. A program, CEEK, was written that used CE to conduct the pair-wise comparison of the query protein structure with every structure in the PDB and generate a list of protein structures ranked by their similarities to the query structure.

Software tools were developed that used the structure based sequence alignments generated by CEEK to analyze the specific sequence and structural motifs of the SH3 domain.

## Supporting Information

Figure S1 **Asymmetric unit of Ack1 kinase domain+SH3 domain structure.** The color coding is the same as in Figure 8, except C-helix is highlighted in cyan.
(TIF)

Figure S2 **Representative ATP titration curves for each Ack1 construct in the non-phosphorylated and phosphorylated forms.** Maximum rate was converted to k_cat_ by dividing the calculated R_max_ by 0.0055 and then by the enzyme concentration in uM. 0.0055 is the calculated extinction coefficient for NAD in the spectromax reader used for all assays using a fill volume of 160 ul per well. Enzyme concentrations used were 12.5 nM for GST-CD and pGST-CD, 100 nM for CD and pCD and 200 nM for KD, pKD, KD+SH3 and pKD+SH3.
(DOCX)

Figure S3 **Representative raw data for an ATP titration using the CD construct.**
(DOCX)
