# Supplementary figures and images for: Ack1: Activation and Regulation by Allostery
Source: PLoS One. 2013 Jan 14;8(1):e53994. doi: 10.1371/journal.pone.0053994 (PMC3544672; doi:10.1371/journal.pone.0053994)

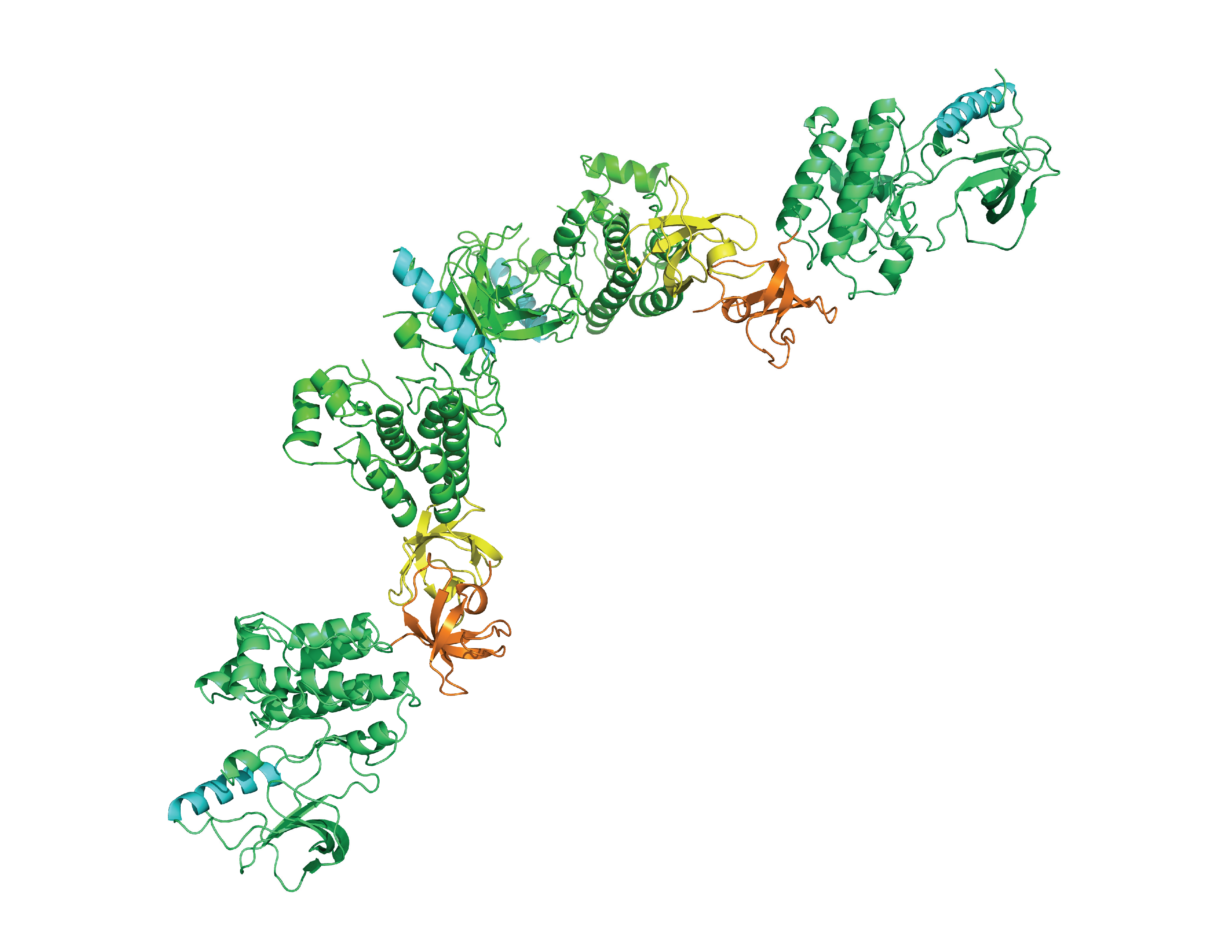

Supplement: Figure S1 — Asymmetric unit of Ack1 kinase domain+SH3 domain structure. The color coding is the same as in Figure 8, except C-helix is highlighted in cyan. (TIF) [file pone.0053994.s001.tif]

Figure S3


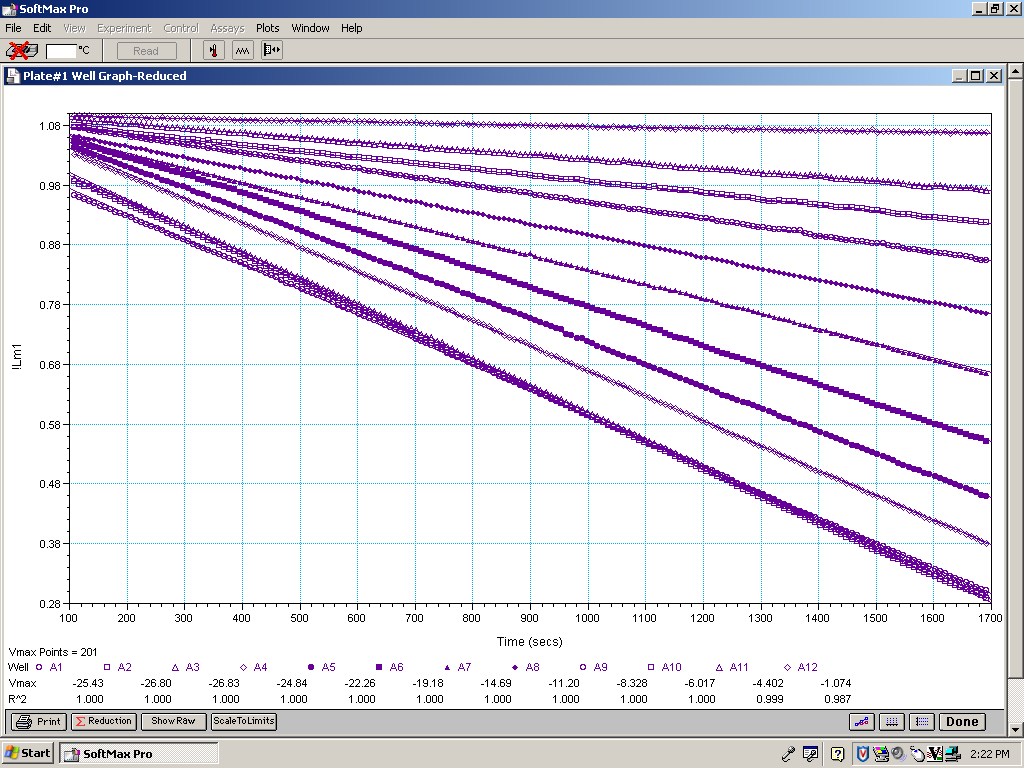

Supplement: Figure S3 — Representative raw data for an ATP titration using the CD construct. (DOCX) [file pone.0053994.s003.docx]
